# Car Safety Airbags Based on Triboelectric Nanogenerators

**DOI:** 10.3390/s26031043

**Published:** 2026-02-05

**Authors:** Bowen Cha, Jun Luo, Zilong Guo, Huayan Pu

**Affiliations:** 1 School of Mechatronic Engineering and Automation, Shanghai University, Shanghai 200444, China; 2 State Key Laboratory of Mechanical Transmission, College of Mechanical and Vehicle Engineering, Chongqing University, Chongqing 400044, China

**Keywords:** triboelectric nanogenerator, automotive airbag, collision sensing, energy utilization, smart car

## Abstract

Triboelectric nanogenerators (TENGs) have gradually been applied in various practical scenarios, mainly focusing on core areas such as wearable motion monitoring devices, medical security systems, and natural resource exploration technology. However, they have the problem of low output energy and have not yet formed effective integration with mature commercially available products, which has hindered the industrialization process. This situation still significantly limits its global promotion and application. In this study, TENG was used as the sensing module for intelligent automotive airbags. We tested the voltage and current output characteristics of the system under different impact forces and frequency conditions. During the testing process, the electrical energy generated under different operating conditions is transmitted to the control system via Metal-Oxide-Semiconductor Field-Effect Transistor (MOSFET) circuits. The system will quickly determine whether to trigger the airbag deployment based on the received electrical signals, and activate the ignition device when necessary to achieve rapid inflation and deployment of the airbag. Compared with traditional triggering mechanisms, the airbag system based on this designed sensor has higher sensitivity and reliability. The sensor can stably capture collision signals, and experiments have shown that as the collision speed increases, the slope of its open-circuit voltage gradually approaches infinity. Applying TENG to automotive airbags not only effectively improves the triggering efficiency and accuracy of airbags, but also provides more reliable safety protection for drivers and passengers. Finite element simulation of the automotive airbag was conducted to provide specific data support for evaluating its safety performance. With the continuous advancement of TENG technology and further expansion of its application scenarios, we believe that such innovative safety technologies will play a more critical role in the future automotive industry.

## 1. Introduction

In recent years, with the rapid development of technologies such as artificial intelligence, the Internet of Things, and big data, smart cars have gradually become the forefront and hotspot of the automotive industry [1,2,3,4,5,6]. Intelligent cars refers to vehicles that achieve highly automated driving by integrating advanced sensors, high-speed computer processors, navigation systems, and complex control algorithms [7,8,9,10,11,12]. This type of car can autonomously perceive the surrounding environment, make decisions, and perform driving tasks, greatly improving driving safety and efficiency. So the core technology of smart cars lies in their sensor networks and data processing capabilities. By processing and analyzing this information through high-speed computer processors, intelligent cars can construct precise environmental models and make decisions in milliseconds for safe driving. Automotive airbags are an important component of intelligent safety systems in cars [13,14,15,16,17,18,19]. They can quickly inflate in the event of a collision, providing additional protection for passengers and reducing the possibility of injury. The importance of airbags lies in their ability to respond to collisions in a very short amount of time and provide additional cushioning for passengers.

The collision sensor is one of the most critical components in airbags. Its main task is to detect whether the vehicle has collided and the severity of the collision. Collision sensors are typically installed at the front and sides of vehicles. When a vehicle collides, the collision sensor senses the impact and converts it into an electrical signal. According to the development history of collision sensors, they are usually divided into two types: electromechanical and electronic. Electromechanical sensors use mechanical motion to control the on/off of airbag circuits [20,21,22]. However, this type of sensor has inherent drawbacks—it cannot distinguish between real collision impacts and false vibrations such as road bumps. Because the inertial force generated by bumps can also drive the steel ball to move, it is prone to misjudgment and false triggering of the airbag, which seriously affects the reliability of the safety system. Due to this critical limitation, electromechanical sensors have gradually been phased out in practical applications. Electronic sensors, by contrast, detect collisions by measuring the changes in the output voltage of capacitors on a microgrid substrate (a core component of microelectromechanical systems, MEMS). The working principle relies on the inertial displacement of the internal mass block caused by collision acceleration, which changes the distance between the capacitor plates and further induces a change in capacitance and output voltage. This design enables electronic sensors to accurately identify collision signals by analyzing the amplitude and duration of voltage changes, effectively filtering out the interference of small-amplitude, short-duration bump vibrations [23,24,25]. Thanks to their high detection accuracy and strong anti-interference ability, electronic sensors have been fully industrialized and widely adopted in mainstream automotive airbag systems at present. Technological innovation is no longer breaking through so quickly. The technology based on TENG has the advantages of light weight, easy manufacturing, low cost, diverse material selection, and simple structure, providing a new strategy for developing airbag sensors [21]. The emergence of new mechanisms and highly sensitive detection sensors often leads to breakthroughs in the TENG field.

Unlike traditional power generation methods, TENG achieves direct conversion from micro-mechanical energy to electrical energy based on the triboelectric effect and electrostatic induction [26,27,28,29,30,31,32,33,34]. The emergence of TENGs not only provides us with a new way of energy collection, but also opens the door to multiple sensing application fields. The application of TENGs in the field of energy has great potential. It can be used as an auxiliary energy source to collect the vibration and friction energy generated during the operation of bicycles, cars, etc., providing continuous and stable power support for some small device equipment [35,36,37]. In the field of environmental science, it can be used to collect mechanical energy in the environment, such as wind energy, water flow energy, etc., and convert it into electrical energy to provide continuous and stable power support for small devices and equipment [38,39,40,41,42]. In the field of biomedicine, its miniaturization allows it to be used to collect tiny mechanical energy within living organisms, such as heartbeats, muscle contractions, etc., to provide energy for implantable medical devices [43,44,45,46]. Although this application technology has advantages in improving energy efficiency, it has not yet shown signs of industrial application. So, combining the equivalent substitution of key components of mature sensors in the market can not only promote the development of the industry, but also often be a pioneer in the development of related disciplines.

In this work, we have developed an easily manufacturable and highly sensitive TENG elastic mechanism, which can be applied to the production of automotive airbags, bringing revolutionary breakthroughs. With its unique principle, it can convert mechanical energy into electrical energy, making the triggering mechanism of airbags more intelligent and efficient, providing passengers with more reliable protection. The electrical output of collision sensors under different impact forces and frequencies is systematically analyzed, with a maximum of 905 V and 56 μA. Subsequently, the feasibility and effectiveness of the design were verified by designing the entire circuit at both ends of the conducting transistor using the results generated by collision. STM32F103ZET6(STM32) can be used to configure a timer to generate signals, which are output to the optocoupler input through GPIO pins. The signal parameters are dynamically adjusted through serial port commands. Comparing the circuits of optocoupler 1 and optocoupler 2, it can be found that the timeliness of optocoupler 2’s circuit reaches a millisecond level. By designing a reasonable control logic, the functions of the central system can be realized, which can trigger the nitroguanide material of the airbag through intelligent algorithms. Due to the characteristics of frictional electricity, collision sensors can achieve ultra-high sensitivity, reaching milliseconds. This work demonstrates the functional application of TENG and can promote its industrialization in the market.

## 2. Results and Discussion

### 2.1. Structural Design

**Figure 1:**
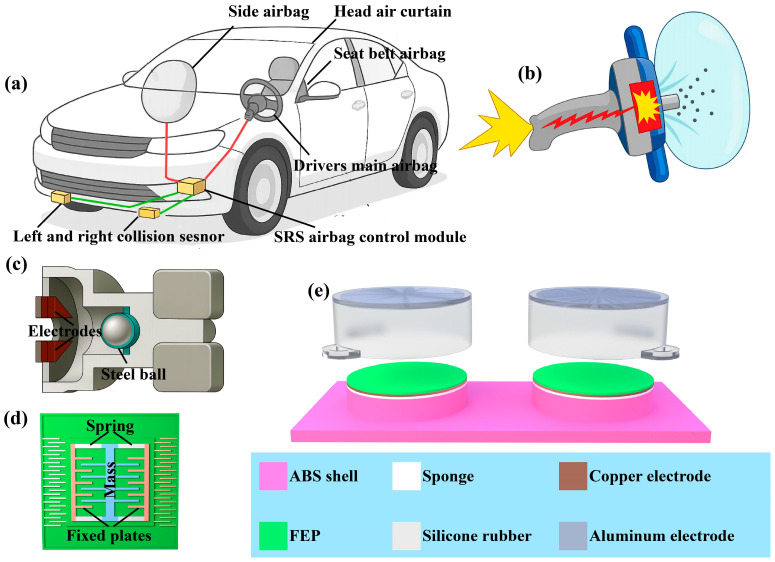
Concept and components of the intelligent system for automotive airbags. (**a**) The installation positions of different types of airbags. The green lines represent the circuit connection between the sensor and control module. The red lines represent the circuit connection between the control block and the airbag. (**b**) Schematic diagram of the gas generator. (**c**) Mechanical collision sensor. (**d**) Accelerometer sensor. (**e**) TENG structural design schematic diagram.

As the core component of passive safety in vehicles, car airbags usually work in conjunction with seat belts to rapidly inflate and cushion the impact force of passengers during collisions, reducing the risk of injury. Figure 1a shows that airbags can be classified into various types based on their installation positions. The driver’s main airbag and front passenger airbag are located in the center of the steering wheel and on the right side of the dashboard, respectively, to prevent the driver’s head from hitting the steering wheel or windshield. Another important feature is the seat belt airbag, which is integrated into the shoulder of the seat belt to relieve pressure on the chest. The head air curtain, distributed along the edge of the car roof, protects the head of passengers during side impact or rollover. In addition, there are knee airbags and rear side airbags (high-end model configurations) to provide additional protection for the driver and rear passengers. The layout of these different airbags aims to cover the critical stress areas during collisions, ensuring that passengers can receive effective protection in various accident scenarios. As shown in Figure 1b, the triggering of the airbag is a rapid response process completed by the cooperation of sensors, control units, and inflation devices. When a vehicle collides, the acceleration and pressure sensors will transmit real-time data to the airbag control unit, which will analyze the collision parameters within a few tens of milliseconds and decide whether to trigger. If the conditions are met, the ignition device will ignite the chemical propellant, producing a large amount of inert gas and rapidly expanding the airbag. Then, the passenger impacts the airbag and releases pressure through the exhaust vent for buffering, which takes about a few hundred milliseconds. In addition, its effectiveness largely depends on the restraint of seat belts, and the deployment of unsecured airbags may actually increase the risk of injury. As posted in Figure 1c, the development history of collision sensors first used steel balls, which can improve the accuracy of the sensor. The steel ball is fixed in place by the attraction of the magnet on the right. When the car collides, the steel ball escapes the attraction of the magnet due to inertia, continues to move forward, and connects the circuit. But the steel ball sensor cannot recognize the situation of bumps. So there are microelectromechanical systems (Figure 1d) that can accurately identify collision situations. But this requires integration with advanced electronic sensors such as speed sensors and gyroscope sensors.

We have designed a flexible TENG to meet the application requirements of collision sensors in automotive airbags, as depicted in Figure 1e. The overall structure of TENG is composed of multiple materials stacked together, mainly including an elastic sponge layer, a copper electrode layer, a fluorinated ethylene propylene (FEP) layer, an aluminum electrode layer, and a flexible encapsulation layer. FEP is a polymer material with excellent insulation properties and frictional electrification characteristics. In the generator, the FEP layer acts as the electron layer and generates charge separation through friction with the aluminum electrode layer. The aluminum electrode serves as the electron loss layer and is in close contact with the FEP layer. The aluminum electrodes have good conductivity and stability, which can effectively transfer charges and maintain the stable operation of the generator. As a supporting structure for generators, flexible substrates require good flexibility, crease resistance, and stability. The flexible substrate material used here is made of silicone. It has good sealing, weather resistance, and chemical stability. The cylindrical shell design of the flexible substrate also ensures a tight fit and stable connection between the materials of each layer. This encapsulation layer also protects the generator to some extent from external environmental influences such as moisture, dust, and mechanical damage. The corresponding physical photographs of the TENG device are provided in Figure S1a,b, and the detailed fabrication process is described in Section 4.

### 2.2. Mechanism of System Modules

**Figure 2:**
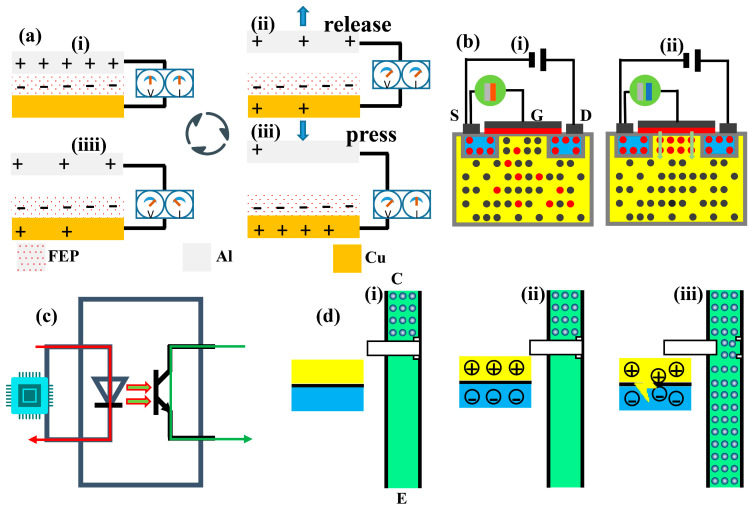
The working mechanism of the system modules. (**a**) Schematic diagram of the working principle of TENG in different states. (**b**) The correlation between MOSFET conduction conditions and TENG. (**c**) Circuit flow of the optocoupler. (**d**) The conduction conditions of the optocoupler.

When a car collides, the structure of the flexible TENG undergoes deformation due to external forces. This deformation causes changes in the contact area and friction degree between the FEP layer and the aluminum electrode layer, leading to a frictional electrification effect. Through electrostatic induction, the electrons on the electrode layer are transferred to the external circuit, generating electrical energy output. When the FEP film comes into close contact with electrodes, electron transfer occurs due to the different electron affinities of the two materials. Usually, electrons transfer from materials with lower electron affinity, such as FEP, to materials with higher electron affinity, such as metal electrodes. This transfer causes the FEP surface to carry a negative charge, while the electrode surface carries an equal amount of positive charge (Figure 2a(i)). At this point, an electrostatic field is formed between the FEP and the electrode. When external mechanical forces separate FEP and electrodes, the electrostatic field between them begins to change. This changing electrostatic field will induce a potential difference in the external circuit (Figure 2a(ii)). To balance this potential difference, electrons begin to flow in the external circuit. Specifically, electrons will flow from the aluminum electrode to the external circuit and then back to the FEP port. The process of electron flow will continue until the FEP and the electrode are completely separated, at which point the electrostatic field reaches a new equilibrium state (Figure 2a(iii)). When the FEP comes into contact with the electrode again, the above process will reverse, and electrons will transfer from the FEP to the aluminum electrode again until a new equilibrium state is reached (Figure 2a(iiii)). By continuously repeating the process of contact and separation, current can be continuously generated in the external circuit.

N-channel MOSFET is a voltage-controlled semiconductor device, whose working principle is based on the regulation of the conductive channel by the gate voltage. MOSFET consists of a P-type substrate, two N +-doped regions (source S and drain D), and a metal-oxide-semiconductor gate (G). A depletion layer will form at the junction of the P-type and N-type semiconductors. When there is no TENG voltage between the gate and source, the diode between the drain and source acts as a back facing diode, making it impossible to conduct, as exhibited in Figure 2b(i). When there is TENG voltage between the gate and source—that is, when the gate source voltage VGS exceeds the threshold voltage VGS(th)—the electric field will repel the holes near the silicon dioxide side of the P-type substrate, causing the immobile negative ion region to form a depletion layer (Figure 2b(ii)). Free electrons will be attracted between the depletion layer and the silicon dioxide layer, forming a conductive channel.

Photocouple is a component that can achieve electrical signal isolation. As displayed in Figure 2c, its input terminal is a light-emitting diode and its output terminal is a phototransistor. When there is no signal input, the light-emitting diode is turned off (Figure 2c(i)). When a signal is input, the light-emitting diode conducts, and the holes in the p-type semiconductor and the electrons inside the n-type semiconductor will gather near the pn junction, which is a process of electronic energy-level transition (Figure 2c(ii)). Electrons transition from a high energy level to a low energy level, and the energy is radiated in the form of photons. This light signal will turn on the phototransistor, and the collector–emitter electrode output terminal will output a signal (Figure 2c(iii)). It can be seen that the optocoupler achieves electrical isolation between input and output signals through the light emitted by the light-emitting diode as a medium.

### 2.3. Output Characteristics of TENG Power

**Figure 3:**
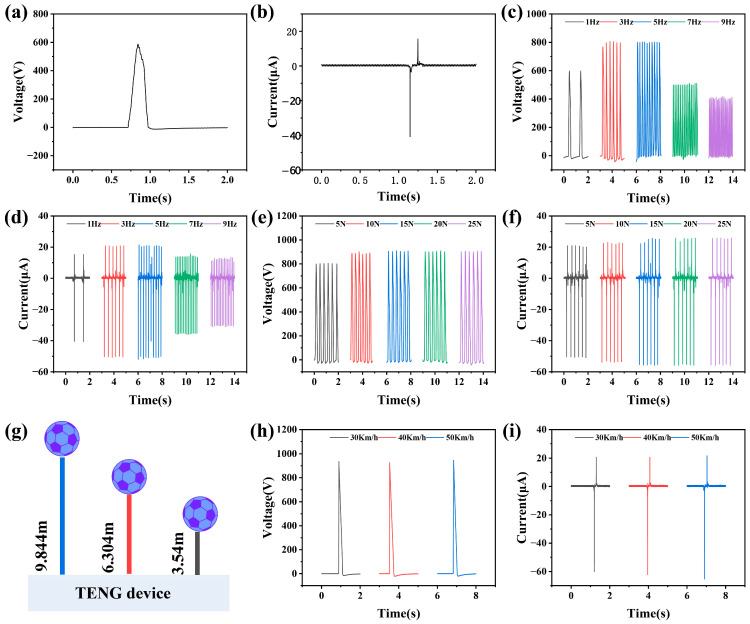
Output characteristics of TENG power. (**a**,**b**) Schematic illustration of forward and backward stimulation application. (**c**,**d**) The open-circuit voltage and short-circuit current of the TENG at different frequencies. (**e**,**f**) The open-circuit voltage and short-circuit current of the TENG at different pressures. (**g**) Schematic diagram of a football undergoing free fall from different heights. (**h**,**i**) The open-circuit voltage and short-circuit current of the TENG at different heights.

In order to qualitatively study the output characteristics of the sensor, a motor was selected as the excitation, and the typical output voltage and current curves of TENG at a frequency of 3 Hz were given. Figure 3a,b show the maximum peak to peak open-circuit voltage of 586 V and the short-circuit current of 46 μA. Based on the analysis of the electronic circuit above, open-circuit voltage and short-circuit current are used as the main parameters to analyze the superiority of the structure and the sensitivity of the material. In order to systematically analyze the influence of sensor output parameters at different frequencies, complex experiments were designed. Figure 3c,d illustrate that the output performance of TENG increases with frequency. When the frequency increases from 1 Hz to 5 Hz, voltage increases from 598 V to 802 V, and current increases from 40 μA to 50 μA. When the frequency increases from 5 Hz to 9 Hz, voltage decreases from 802 V to 410 V, and current decreases from 50 μA to 30 μA. The results indicate that both voltage and current first increase with frequency and then decrease with frequency after exceeding 5 Hz. This is because the elasticity of the silicone shell has not yet unfolded, resulting in a decrease in the distance between the FEP and the electrode. When subjected to the mechanical pressure of different strengths, the elastic modulus, hardness, toughness, and other properties of the silicone film structure are prone to deformation, which affects the output of TENG sensors. Therefore, it is necessary to analyze the influence of sensor output parameters under different pressures and design corresponding experiments. Figure 3e,f indicate that as the pressure increases from 5 N to 15 N, the voltage increases from 803 V to 905 V and the current increases from 50 μA to 56 μA. When the pressure increases from 15 N to 25 N, the voltage and current remain unchanged.

To verify the output performance of the TENG at different collision speeds, a set of free fall impact experiments is designed. The experiment controlled the free fall height of the football to achieve speeds of 30 km/h, 40 km/h, and 50 km/h when it collided with the TENG contact separation mode device, and then measured the output voltage and current signals of the generator under different impact conditions. According to the free fall formula, the required release heights are calculated as follows: 30 km/h (8.33 m/s) corresponds to 3.54 m, 40 km/h (11.11 m/s) corresponds to 6.3 m, and 50 km/h (13.89 m/s) corresponds to 9.84 m. During the experiment, the TENG is fixed at the bottom of the falling platform, and the football is released freely from the predetermined height in sequence, as exhibited in Figure 3g. The voltage and current output signals of the TENG are recorded by an electrostatic meter. Analyze the influence of collision speed on the output performance of TENG, and provide experimental evidence for their application in automotive collision detection systems. As shown in Figure 3h, as the collision speed increases, the peak output voltage increases from 935 V at 30 km/h to 946 V at 50 km/h, and the voltage remains stable. However, the slope of the voltage rise here shows a strong linear correlation with the collision speed. Regardless of whether it is 30 km/h or 50 km/h, the slope of the voltage quickly reaches its maximum value during extreme times. Meanwhile, the current output characteristics are difficult to use as a dependence on car speed and cannot reflect the changes in collision over time, as displayed in Figure 3i. Experimental data confirms that the voltage output slope of TENG sensors can serve as a reliable indicator of collision speed. Specifically, within different speed ranges, the speed of the voltage rise slope can effectively distinguish the speed of the conducting N-type MOSFET, providing a new technological approach for intelligent triggering decisions of airbags. These findings not only validate the applicability of TENG in automotive passive safety systems, but also provide an important basis for the optimization design of next-generation self-powered collision sensors based on the revealed dynamic response laws.

### 2.4. Application of Automotive Airbags Under the Verification and Selection of Optocouplers

**Figure 4:**
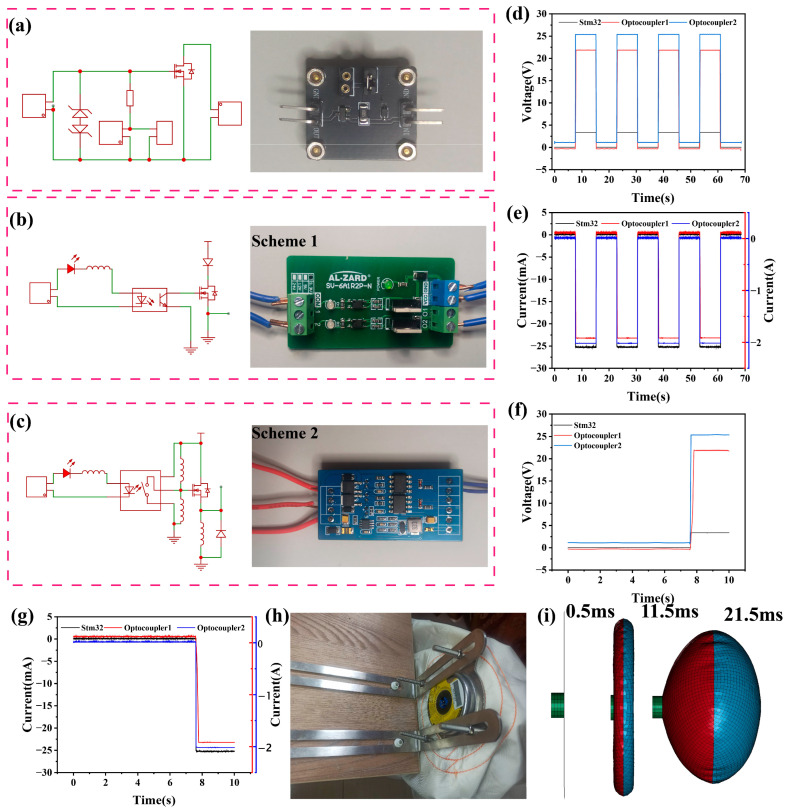
Application demonstration of TENG in car collision detection. (**a**) The MOSFET conduction circuit and physical diagram of the Teng sensor. (**b**,**c**) Circuit diagrams and physical images of optocoupler 1 and optocoupler 2. (**d**,**e**) Macro comparison diagram of voltage and current for stm32, optocoupler 1, and optocoupler 2. (**f**,**g**) Microscopic comparison diagram of voltage and current for stm32, optocoupler 1, and optocoupler 2. (**h**) Test bench for airbag installation. (**i**) The simulated dynamic process of the airbag.

This study designed an intelligent collision sensor system based on the contact separation mode of a TENG. By combining the efficient mechanical to electrical energy conversion characteristics of TENG with the fast-switching function of MOSFET tubes, precise control of airbag triggering signals was achieved. The gate input impedance of MOSFET is very high, making it difficult to release induced charges. The high voltage generated can easily break down the thin insulation layer, causing permanent damage to the MOSFET, because after being broken down, the charge can no longer accumulate to form an N-channel. TENG generates instantaneous high-voltage pulses due to contact separation when impacted, which are directly applied to the gate of the N-channel MOSFET. When the voltage far exceeds the threshold voltage of the MOSFET, it is found that excessive voltage can cause damage to the MOSFET. As shown in Figure 4a, the characteristics of TENG are high voltage and low current, and by adding the TVS transistors, it is easy to reach the contact spring without damaging the MOSFET. At the same time, in order to increase the sensitivity of the circuit, a pluggable resistor channel was designed. When a conductive channel is formed between the drain and the source, a high-level signal is transmitted to the General-Purpose Input/Output (GPIO) detection pin of the STM32 microcontroller. STM32 monitors the level changes in this pin in real-time, determines the occurrence of collision events, and outputs a trigger signal to the airbag ignition circuit to complete the rapid detonation of the airbag. This system utilizes the self-powered characteristics of TENG to achieve collision detection without the need for an external power source, and has the advantages of high efficiency, environmental friendliness, and sustainability. At the same time, the fast response (microsecond level) of MOSFET ensures real-time signal transmission, while the software logic of STM32 can further optimize the filtering algorithm for false triggering, improving the reliability of the system.

This study designed a dual optocoupler circuit testing scheme based on an STM32 control board to meet the real-time requirements of airbag triggering systems. By comparing the response characteristics of millisecond-level optocouplers (scheme 1) (Figure 4b) and microsecond-level optocouplers (scheme 2) (Figure 4c), the system response time was optimized. The experiment uses the STM32 series controller to generate frequency adjustable high- and low-level signals (0 and 3.3 V), which are, respectively, input to the input terminals of two optocoupler circuits (LK36345T millisecond optocoupler and 6N137 microsecond optocoupler). The output terminal of the optocoupler is connected to the simulated load circuit of the airbag, and captures the timing waveform of the input trigger signal and the optocoupler output signal in real time. Adjust the duty cycle and frequency of the STM32 output pulse, set the time difference to 7.2 s, and then test the conduction delay time and rising/falling edge time of the two optocouplers under different operating conditions and record the time difference measured by the electrostatic meter.

As illustrated in Figure 4d,e, the experimental results demonstrate that from a macroscopic perspective, the response times of optocoupler 1 and optocoupler 2 are consistent with the input time sequence of the control chip (in seconds). However, the automotive airbag system demands an ultra-fast response to ensure passenger safety in collision accidents. Therefore, the voltage and current signals extracted from a microscopic perspective (Figure 4f,g) reveal that compared with the millisecond-level response of optocoupler 1, the response time of the microsecond-level optocoupler 2 is reduced by one order of magnitude, which can significantly improve the triggering speed of the airbag system and provide experimental data support for optocoupler selection in practical applications. For the ignition of the gas generator using guanidine nitrate as the propellant, the magnitude of the trigger current is a critical parameter, which requires a minimum current of 1 A to initiate the combustion reaction, while the stable trigger current for reliable ignition is 1.5 A. It should be emphasized that Figure 4g employs dual vertical axes for current characterization, which is designed to clearly present the current signals of components with distinct magnitude differences in a single plot, and the detailed design principle of the axes is elaborated as follows: the left vertical axis is calibrated in milliampere (mA) scale, corresponding to the working current of the STM32 control chip. As a low-power microcontroller, STM32 only operates at a current level of several to tens of mA to output collision control signals, which is far lower than the current required for gas generator ignition. In contrast, the right vertical axis is calibrated in ampere (A) scale, representing the trigger current of optocoupler 1 and optocoupler 2. The current of the optocouplers is supplied by the vehicle power supply (bench tests in this work adopted household-level power supply), which is sufficient to drive the gas generator.

As shown in Figure 4h and Figure S2a, the airbag gas generator is the core component of the airbag system, responsible for quickly generating a large amount of gas at the moment of collision, causing the airbag to inflate and deploy in milliseconds. The structural design directly determines the deployment speed, gas volume, and safety of the airbag. As exhibited in Figure S2b, the gas generator consists of a metal casing, ignition device, chemical propellant, and gas diffusion chamber. The metal casing is equipped with precise gas discharge holes to control the direction of airflow, and the ignition device ignites the propellant through an electric heating wire. Sodium azide (NaN_3_) is a commonly used gas generator in traditional automotive airbags, but its chemical properties are active and have certain toxicity, posing safety hazards in production, storage, and handling processes [47,48,49]. As an environmentally friendly alternative to sodium azide, guanidine nitrite (CH_6_N_4_O_3_) not only rapidly decomposes and produces gas when the car airbag is triggered, meeting safety protection requirements, but also gradually becoming an important choice for green technology upgrading in the field of car safety due to its more stable chemical properties and lower environmental hazards [50,51,52,53,54]. Its explosive decomposition reaction is a complex redox process involving the generation of multiple gas products. The thermal decomposition of guanidine nitrate mainly produces gases through the following pathways.

**3CH_6_N_4_O_3_→3CO_2_↑ + 6H_2_O↑ + 9N_2_↑** (1)

Additionally, 100 g of guanidine nitrate can generate approximately 50 L of gas (under standard conditions) to meet the inflation requirements of the airbag. At the same time, in the presence of high temperature or catalyst, the following side reactions may occur.

**CH_6_N_4_O_3_→HCNO↑ + NH_3_↑ + N_2_O↑**(Trace toxic by-products) (2)

The control measure is to add copper oxide (CuO) or iron oxide (Fe_2_O_3_) as catalysts to suppress the generation of HCNO.

As shown in Figure S3, connect the TENG–MOSFET module to the STM32 control board, optocoupler circuit, and airbag. Conduct a small-scale simulated real vehicle collision test to observe the triggering success rate and response time of the system, from the occurrence of the collision to the STM32 output signal, and then to the explosion of the airbag. From Video S1 and Figure S2c, it can be seen that the experimental results show that the integrated system exhibits excellent performance under simulated collision conditions. Within the collision speed range of the car sliding, the system triggers successfully with a response time in milliseconds. This experiment uses simulation modeling methods to reproduce the deployment process of airbags in collision situations. As shown in Figure 4i, firstly, a safety airbag made of initially flat fabric, the gas generator, is triggered at the beginning of the simulation (t = 0). The gas generator sprays gas into the airbag according to the properties defined by the airbag. The gas pushes the fabric airbag to deploy and gradually expand. The detailed simulation process can be seen in Section 4 and Video S2. Figure S4a shows three nodes on the airbag for numerical displacement and velocity analysis. As displayed in Figure S4b,c, the simulation results indicate that the airbag can respond promptly and fully deploy under the set conditions, and its buffering displacement and inflation speed meet the actual needs. Figure S5a shows three elements on the airbag for numerical pressure analysis. Meanwhile, as exhibited in Figure S5b, it was also found in the simulation that the timing of airbag deployment and inflation pressure have a significant impact on the protection effect, indicating that further optimization needs to consider sensor sensitivity and inflation control strategies. These data fully demonstrate the feasibility and technical advantages of integrating TENG technology into automotive airbag systems, not only improving the overall reliability of the system, but also providing a new technological path for the development of future intelligent airbag systems. The testing methods and technical parameters established in this study have laid an important experimental foundation for subsequent industrial applications.

### 2.5. Application of IoT Alarm

**Figure 5:**
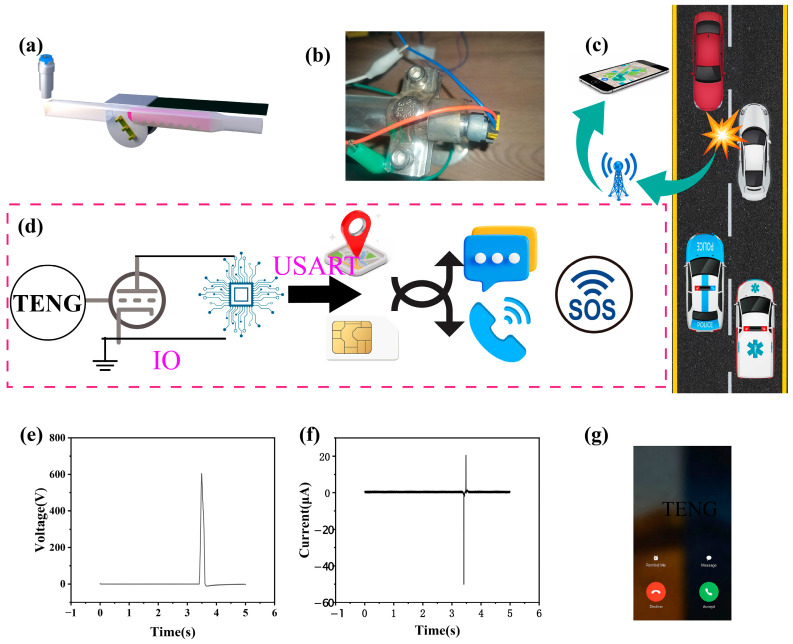
A demonstration of Teng application in the Internet of Things (IoT) used for directly displaying and remotely monitoring critical information. (**a**) Structural diagram of the seat belt. (**b**) Setup of the experiment platform of the seat belt (**c**) The signal flow of the designed device in actual application scenarios. (**d**) System indication diagram for remote monitoring and direct display of GPS positioning. Used to display the flow of information conversion. (**e**,**f**) Voltage and current diagram of car collision with TENG equipment. (**g**) Automatic triggering of preset call alarm schematic diagram.

As shown in Figure 5a, when a collision occurs, the TENG trigger signal activates the ignition device within milliseconds, igniting the main charge to produce high-pressure gas, breaking through the aluminum object, and pushing the piston to eliminate an appropriate amount of seat belt slack. The relevant internal structure is shown in Figure S6a,b. The entire process is controlled to be completed within a few tens of milliseconds. Compared to the main airbag generator, the tensioner generator has a faster response speed and lower working pressure. The simplified testing platform is exhibited in Figure 5b. The gas generator after the explosion can be seen in the Video S3 and Figure S6c.

The high-sensitivity TENG sensor can detect various collision accidents in real time, including low-speed collisions and high-speed severe collisions, solving the problem of missed reports of traditional sensors in various collision scenarios. Adopting self-powered TENG sensors eliminates dependence on onboard power sources. In addition, it reduces the false alarm rate through MOSFET hardware filtering and the STM32 debounce algorithm. With the development of intelligent connected vehicles, integrated safety solutions will become a standard for future vehicles, not only improving the safety performance of individual vehicles, but also helping to build an efficient road accident emergency response network, as shown in the drawing in Figure 5c. A timely and accurate automatic alarm can also reduce the incidence of secondary accidents and overall accident mortality rate.

This study designed an intelligent safety system that integrates collision detection and location alarm. The system adopts a contact separation TENG, combined with an MOSFET switch circuit and STM32 microcontroller, to achieve multi-level signal processing, as shown in Figure 5d. When the output voltage of TENG exceeds the threshold voltage of the MOSFET, drain source conduction occurs. When STM32 detects a signal, it sends specific information through the serial port. Here, a commercial GPS positioning alarm module and SIM800L are used, as shown in Figure S7a,b. The voltage and current generated during a car collision are shown in Figure 5e,f. After detecting a valid collision signal, immediately read the latitude and longitude data, and automatically trigger the preset voice call and text message alarm, as shown in Figure 5g and Figure S7c. Video S4 shows that the entire process from collision occurrence to alarm information transmission is controlled within 60 s, including millisecond-level collision confirmation time, millisecond-level positioning stability time, and second-level communication establishment time. This rapid emergency response capability enables emergency personnel to quickly arrive at the scene. This study innovatively integrates the high sensitivity and reliability characteristics of TENG with IoT technology, opening up a new technological path for vehicle accident automatic alarm systems. TENG sensors, with their unique contact separation working principle, achieve the precise detection of small collisions. Their subtle response speed breaks through the technical bottlenecks of traditional sensors in terms of sensitivity and energy consumption. Through system integration with IoT technology, a closed-loop security protection system integrating collision perception, precise positioning, and intelligent alarm was constructed. This innovative paradigm of integrating new energy sensing technology with intelligent IoT not only provides better solutions for vehicle safety systems, but its technical framework can also be extended to industrial equipment monitoring, infrastructure safety warning, and other fields, demonstrating broad application prospects and industrial value.

## 3. Conclusions

This work proposes and investigates an intelligent collision sensor system based on the TENG contact separation mode principle, whose sensing mechanism has the characteristics of high efficiency, environmental protection, and sustainability. In addition, it also has the advantages of facile fabrication, easy manufacturing, and high integration. TENG can convert the mechanical energy generated by car collisions into electrical energy, providing a stable and reliable source for triggering airbags. Through system experiments, it was verified that the designed TENG device can generate an open-circuit voltage of 905 V under the combined action of an excitation frequency of 3 Hz and an external force of 15 N. From the perspective of circuit operation requirements, the output voltage has far exceeded the conduction voltage requirement of the MOSFET, and can stably achieve reliable conduction of the MOSFET, providing sufficient voltage guarantee for subsequent energy transmission or signal control links. The intelligent control system can use the potential difference between the drain and source of the transistor to detect the collision responses, which can effectively identify signals with good accuracy. The TENG–MOSFET module is connected to the STM32 control board, optocoupler circuit, and airbag. Through a small-scale simulated automobile collision test, it successfully ignited guanidine nitrate, which has high reliability and real-time response time at the millisecond level. An intelligent safety system integrating collision detection and positioning alarm was constructed, which can send latitude and longitude data to a set mobile phone number through SMS and automatically trigger preset call alarms. The application of TENG in automotive airbags has significant advantages and potential. It not only improves the triggering efficiency and reliability of airbags, but also brings new technological breakthroughs to the field of automotive safety. With the deepening of research and further development of technology, the structure, signal recognition methods, and core application scope of sensors for automotive airbags can be further improved in future work, providing passengers with more comprehensive and efficient protection. In subsequent research, we will conduct a comprehensive comparative analysis of the proposed triboelectric collision sensor with traditional commercial micromachined acceleration sensors in multiple performance dimensions.

## 4. Experimental Section

Construction of contact separation mode TENG: To prepare TENG, we chose FEP thin film (thickness 0.1 mm, commercially available, with no additional surface modification before use) with strong electron acquisition ability as the solid contact surface. In order to process the induction electrode on the back of the FEP, we directly pasted the copper electrode (thickness 0.06 mm, commercially available, with no additional surface modification before use) onto the FEP film. We chose an aluminum film (thickness 0.06 mm, commercially available, with no additional surface modification before use) with strong electron acquisition capability as the other contact surface of the solid. Finally, connect and fix the manufactured FEP device with wires onto the PLA material printed substrate (with a diameter of 5.8 cm, Model [PLA-175], diameter 1.75 mm, commercially available). A layer of sponge (thickness 2 mm, commercially available, with no additional surface modification before use) was pre-applied on top of it. Another aluminum electrode is attached to silicone gel (with a diameter of 6 cm, commercially available, used for structural bonding and encapsulation of the TENG device).

Characterization and electrical measurements: The short-circuit current and open-circuit voltage were examined through a Keithley 6514 electrometer.

Testing of optocoupler circuit: The optocoupler experimental testing system consists of three parts: the signal generation module, optocoupler testing module, and data acquisition module. Firstly, use STM32 to configure a timer to generate a signal, which is output to the optocoupler input through GPIO pins. The signal parameters are dynamically adjusted through serial port commands. The optocoupler testing module adopts an independent power supply design. The data acquisition module asynchronously captures the STM32 output signal and optocoupler output signal through an electrostatic meter, and then organizes them. This experiment verifies the necessity of high-speed optocouplers in airbag systems and provides optimization basis for the timing design of STM32 control circuits.

Automotive collision function testing platform: A vehicle simulation platform was established on a template platform and validated under two typical operating conditions: (1) Under the optocoupler 2 scheme, the airbag exploded within a few milliseconds after a collision. (2) In the forward collision, under the optocoupler 2 scheme, the gas generator on the seat belt exploded within a few milliseconds after the collision.

Automotive collision IoT alarm platform: Forward collision—the system needs to complete GPS positioning after the collision and issue a call and SMS alarm within 60 s. The GPS used is GPS (NEO-6M), and the networking module is SIM800L GSM/GPRS.

Simulation software and environment for airbags: The simulation platform is LS-DYNA. Post-processing is LS-PrePost (result analysis). The parameters of the airbag unit can be found in Figure S8 and Table S1.

## Data Availability

The original contributions presented in this study are included in the article/Supplementary Material. Further inquiries can be directed to the corresponding author(s).

## Supplementary Materials

The following supporting information can be downloaded at: https://www.mdpi.com/article/10.3390/s26031043/s1, Figure S1: The corresponding photographs of the TENG device; Figure S2: Structure diagram of the gas generator; Figure S3: Photograph of the experimental set-up; Figure S4: Dynamic response of the airbag during deployment; Figure S5: Finite element simulation results of the airbag deployment process; Figure S6: The corresponding photographs of gas generator for automotive airbag; Figure S7: System hardware composition and alarm effect display.; Figure S8: Simulation parameters of airbag unit in LS-DYNA software; Table S1: The descriptions of airbag elements used in this work; Video S1: Explosion moment of gas generator in airbag; Video S2: Finite element simulation of safety airbags; Video S3: Explosion of gas generator in seat belt; Video S4: Display of Internet alarm in case of vehicle collision.
