# Supplementary material for: Car Safety Airbags Based on Triboelectric Nanogenerators
Source: Sensors (Basel). 2026 Feb 5;26(3):1043. doi: 10.3390/s26031043 (PMC12900148; doi:10.3390/s26031043)
Supplement: Supplementary file 1 [file sensors-26-01043-s001.zip › Supporting Information.pdf]

Supporting Information

Car Safety Airbags Based on Triboelectric Nanogenerators

Bowen Cha [1,*], Jun Luo [2], Zilong Guo [1] and Huayan Pu [1]

[1] School of Mechatronic Engineering and Automation, Shanghai University, Shanghai 200444, China; guozl@shu.edu.cn (Z.G.); phygood_2001@shu.edu.cn (H.P.)
[2] State Key Laboratory of Mechanical Transmission, College of Mechanical and Vehicle Engineering, Chongqing University, Chongqing 400044, China; luoj@cqu.edu.cn
* Correspondence: chabowen@shu.edu.cn

Supporting Videos

Supporting Video S1. Explosion moment of gas generator in airbag.

Supporting Video S2. Finite element simulation of safety airbags.

Supporting Video S3. Explosion of gas generator in seat belt.

Supporting Video S4. Display of Internet alarm in case of vehicle collision.

Supporting Tables

Supporting Table S1. The descriptions of airbag elements used in this work

Parameter	Description	Numerical value	Unit
R(out)	Outer diameter	300	mm
R(in)	Inner diameter	40	mm
ρ	Rigid density	7.84e-6	Kg/mm³
E	Elastic modulus	200	GPa
ν	Poisson's ratio	0.3	(-)
ρ	Fabric density	8.76e-7	Kg/mm³
EA	Longitudinal elastic modulus	0.3	GPa
EB	Latitudinal elastic modulus	0.2	GPa
PRBA	Poisson's ratio of meridional to latitudinal direction	0.2	(-)
PRAB	Poisson's ratio of latitude to longitude	0.2	(-)
GAB	In-plane shear modulus	0.04	GPa
CSE	Yield stress constant	1	(-)
EL	ultimate strain	0.06	(-)
PRL	Ultimate poisson's ratio	0.35	(-)
DAMP	Damping coefficient	0.2	(-)
CV	Constant-Volume specific heat	196.168	mJ/(kg·K)
CP	Constant-Pressure specific heat	274.59	mJ/(kg·K)`
T	Inflation temperature	800	K
MU	Vent coefficient	0.7	(-)
PE	Ambient pressure	1.013e-4	GPa
RO:	Ambient density`	1.225e-9	Kg/mm³

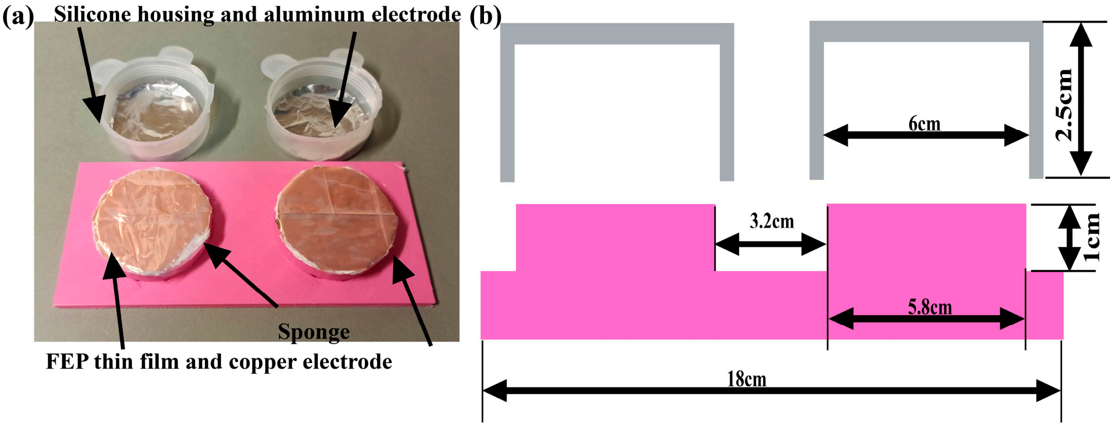

Figure. S1. The corresponding photographs of the TENG device. (a) 3D assembly diagram of the TENG sensor. (b)The structure dimension.

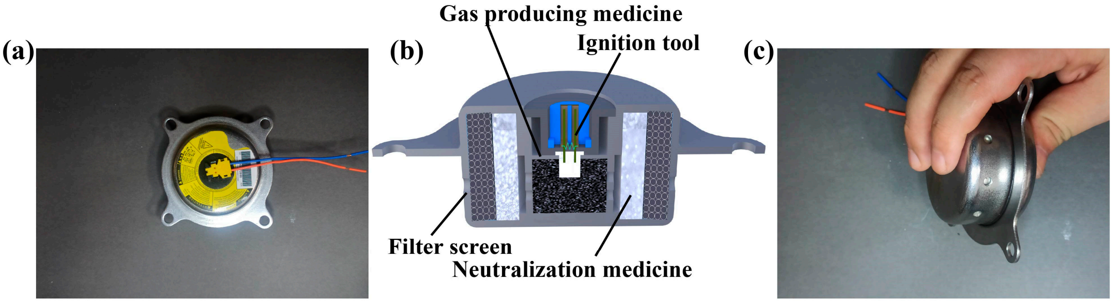

Figure. S2. Structure diagram of the gas generator. (a) Photograph before explosion. (b) Internal architecture of gas generator. (c)Photograph after explosion of gas generator.

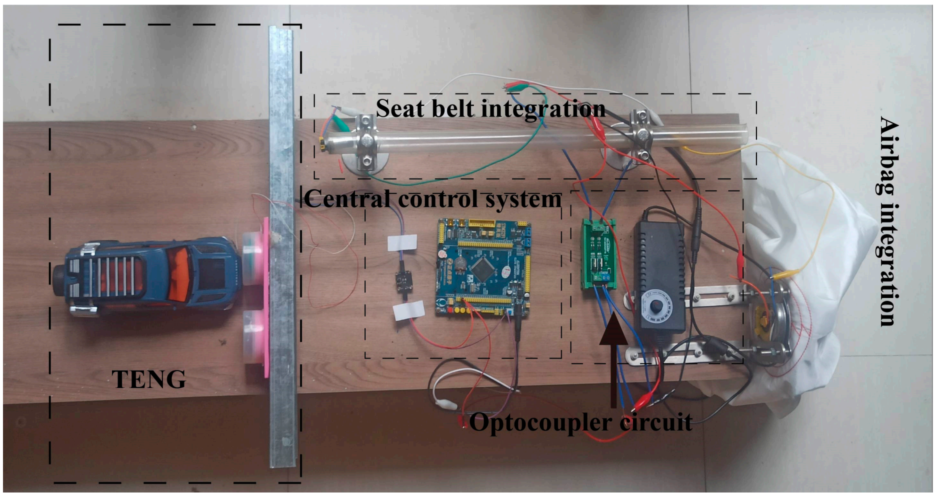

Figure. S3. Photograph of the experimental set-up.

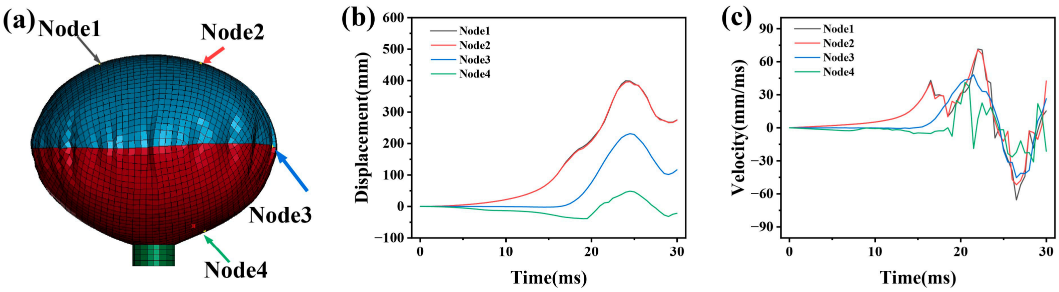

Figure. S4. Dynamic response of the airbag during deployment. (a) Definition of the three representative nodes (Node A, B, and C) on the airbag model. (b) Temporal evolution of nodal displacements. (c) Temporal evolution of nodal velocities, indicating the peak contact velocity.

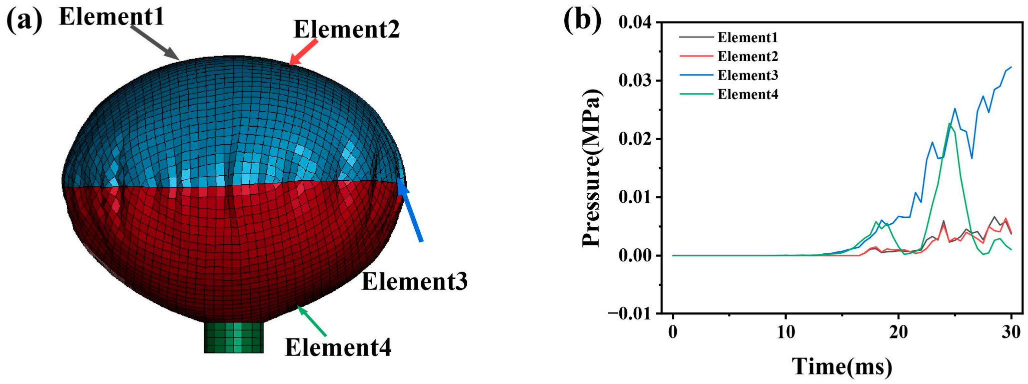

Figure. S5. Finite element simulation results of the airbag deployment process. (a) Locations of the three monitored elements on the airbag. (b) Pressure-Time history of the monitored nodes.

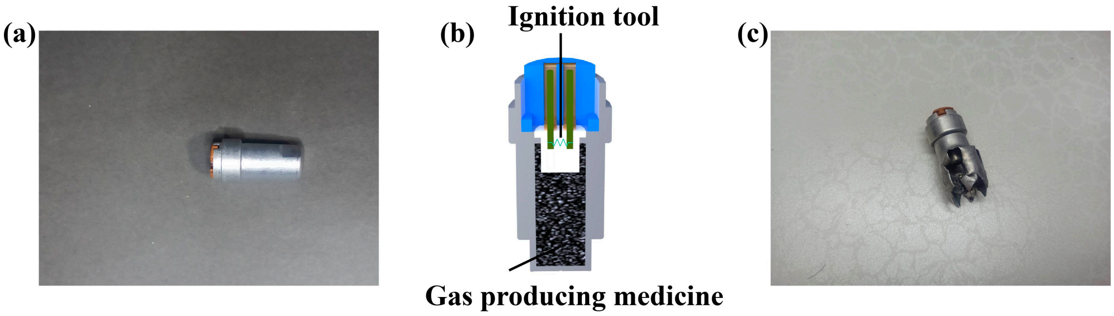

Figure. S6. The corresponding photographs of gas generator for automotive airbag. (a) A complete gas generator. (b) Internal architecture of gas generator. (c)Gas generator after blasting.

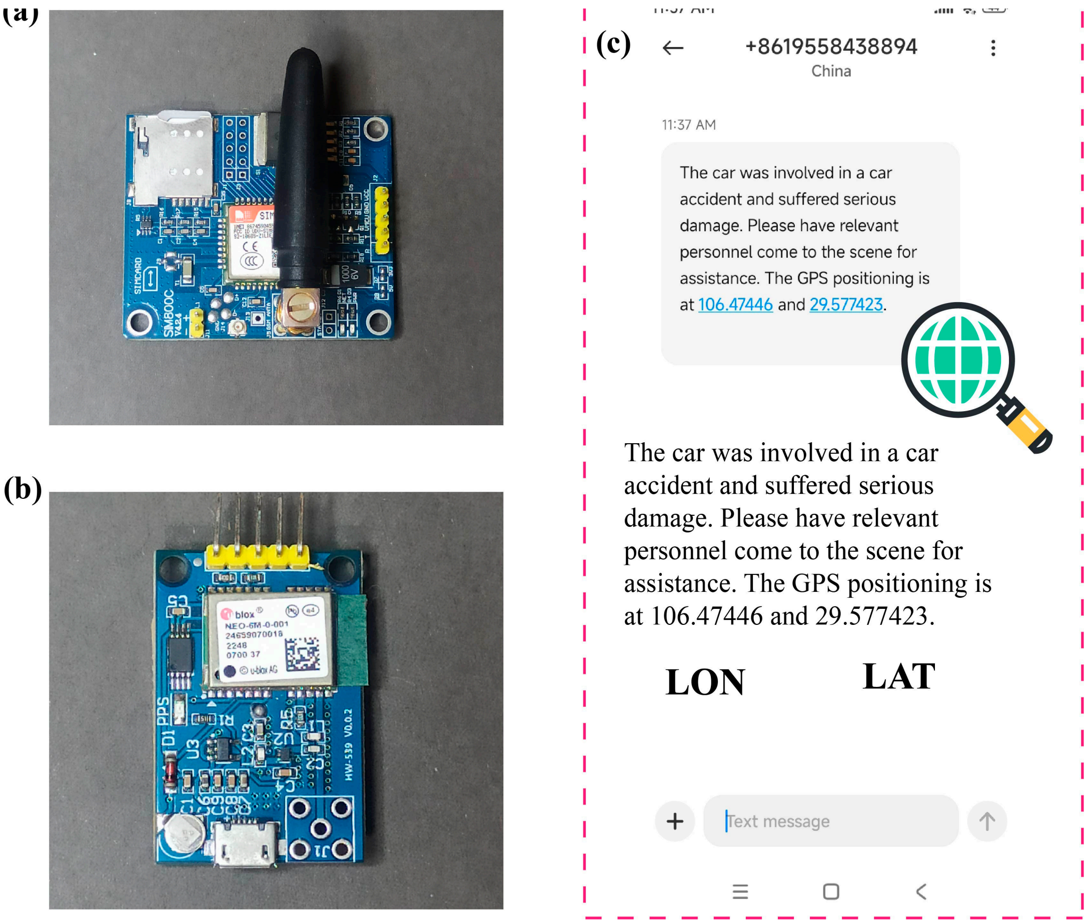

Figure. S7. System hardware composition and alarm effect display. (a) SIM800C GSM/GPRS module for base station communication. (b) GPS(NEO-6M) positioning module for capturing geographical locations. (c) SMS of GPS positioning information received by the mobile terminal.

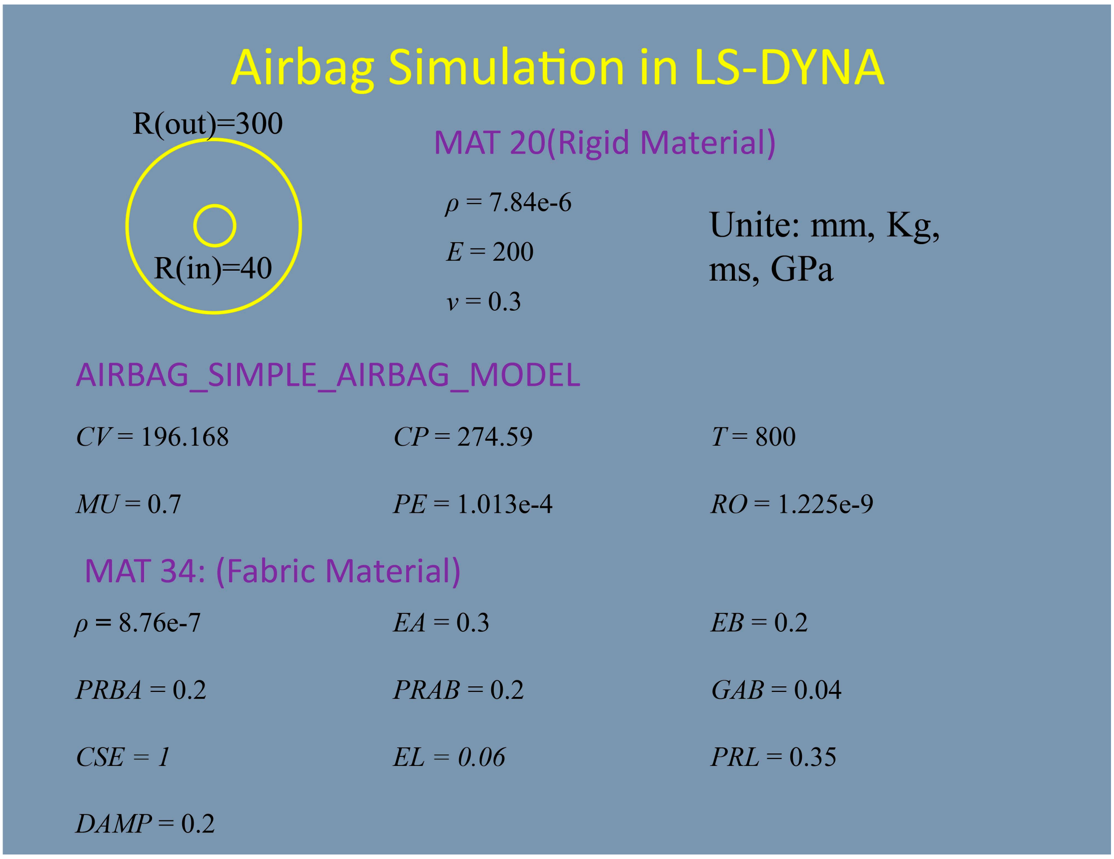

Figure. S8. Simulation parameters of airbag unit in LS-DYNA software.
